# L-Lysine-grafted graphene oxide as an effective adsorbent for the removal of methylene blue and metal ions

**DOI:** 10.3762/bjnano.8.268

**Published:** 2017-12-13

**Authors:** Yan Yan, Jie Li, Fangbei Kong, Kuankuan Jia, Shiyu He, Baorong Wang

**Affiliations:** 1 College Of Science, North China University of Science and Technology, Tangshan 063000, China; 2 Engineering Computing and Simulation Innovation Laboratory, North China University of Science and Technology, Tangshan 063000, China; 3 Ministry of education key laboratory with modern metallurgical technology, North China University of Science and Technology, Tangshan 63000, China

**Keywords:** adsorption, copper (Cu) ions, graphene, isotherms, methylene blue

## Abstract

In this paper, novel L-lysine-modified graphene oxide (Lys-GO) was synthesized through amidation. The morphological and structural properties of Lys-GO were characterized using infrared spectrometry, scanning electronic microscopy and X-ray photoelectron spectroscopy. The as-prepared Lys-GO material was systematically investigated in a series of batch adsorption experiments for the removal of methylene blue (MB) and copper ions (Cu^2+^) from wastewater. These results showed that Lys-GO is a bifunctional adsorbent for the removal of dyes and metal ions, and excellent adsorption efficiency was obtained. The maximum adsorption capacities for MB dye and Cu^2+^ were 1679.1 mg/g and 186.9 mg/g at 35 °C, respectively. The kinetics of adsorption followed well the linear pseudo-second-kinetic model. The isotherm results indicated that MB adsorption can be described with the Langmuir isotherm model, while the adsorption of Cu^2+^ can be described with the Freundlich model. The excellent adsorption capacity indicated that the Lys-GO may be a promising adsorption material for the removal of environmental pollutants.

## Introduction

Graphene is a two-dimensional carbon material with honeycomb network and sp^2^ hybridization. Recently, graphene-based materials have drawn attention because of their huge specific surface area, extraordinary electronic transport properties and unique adsorption properties [1–3]. These materials have important applications in many fields, including physics [4], electrochemistry [5], environmental science [6] and catalysis [7]. For example, a MoS*_x_*/3D-graphene hybrid material as an electrode material enhanced the efficiency of hydrogen-producing in a fuel cell [8]. Mo et al. reported reduced graphene oxide covalently functionalized with L-lysine [9], which could be used for the electrochemical recognition of tryptophan (Trp) enantiomers. Reduced graphene oxide as an effective adsorbent can be used for the removal of malachite green dye and metal ions [10–11]. A high-performance hydrophilic polyvinylidene fluoride/graphene oxide (PVDF/GO)–lysine composite membrane can be used for sea water desalination and purification [12]. However, the strong cohesive interactions of graphene basal planes and edges have also caused some difficulties in attaining its optimal performance. Hence, the functionalization of graphene has been extensively developed to further improve its properties and broaden its potential application, such as loading of organic molecules and metal nanoparticles via covalent or non-covalent binding [13–16].

Recently, functionalized graphene materials have shown great potential as highly efficient absorbers for the treatment of environmental pollutants and wastewater purification [17–19]. However, most of the functionalized graphene materials cannot meet practical needs in treating environmental pollutants because of high cost and low performance. Hence, the adsorption performance of graphene-based materials still needs to be improved and the cost lowered. Some reports showed that oxygen functional groups, vacancy defects and π–π interactions on the graphene basal planes and edges can enhance the adsorption capacities for pollutants [3,20]. We inferred that organic molecules modifying graphene might improve their adsorption capabilities. For instance, Swager et al. reported the surface functionalization of graphene oxide (GO) with malononitrile can increase the solubility in either organic or aqueous environments [21]. However, no practical application for the malononitrile-modified GO was described. Interestingly, Ma et al. reported an polyethyleneimine-functionalized ultra-light graphene aerogel (PFGA) used as an adsorbent for the removal of methyl orange and amaranth in 2017 [22]. The maximum adsorption capacities of PFGA for methyl orange and amaranth were as high as 3059.2 mg/g and 2043.7 mg/g, respectively. Herein, we inferred that grafting amino groups onto graphene might enhance the performance of graphene materials.

According to this analysis, we tried to synthesize a novel graphene material for the removal of heavy metal ions and organic dyes from wastewater. In 2013, Gao et al. developed an environmentally friendly approach to reduce GO with L-lysine [10], which showed potential applications for the removal of metal ions from wastewater. However, L-lysine failed to be grafted onto the reduced graphene oxide (RGO). Results showed a slightly lower absorbing capacity for copper ions (Cu^2+^).

Herein, L-lysine was attached to the surface of GO by amidation between –COOH and –NH_2_ to form Lys-GO. GO is an acidic material, and the basic L-lysine can adjust the acid–base properties of GO. Lys-GO showed excellent adsorption capability for the removal of methylene blue (MB) and Cu^2+^ from simulated wastewater.

## Results and Discussion

### Characterization of the Lys-GO hybrid

**Figure 1:**
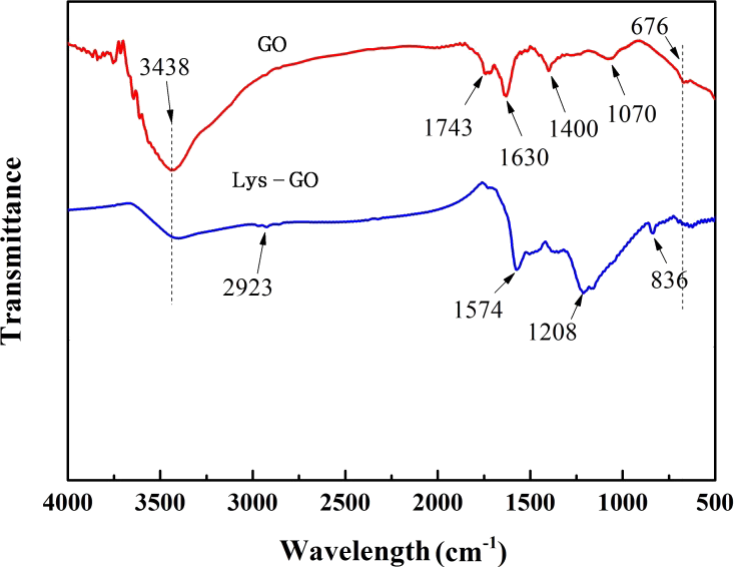
FTIR analysis of the obtained GO and Lys-GO.

Firstly, the obtained materials were characterized by FTIR analysis (Figure 1). For GO, aromatic C=C and C=O stretching vibrations can be clearly seen at ca. 1630 and 1743 cm^−1^. The strong peaks around 3438 and 1400 cm^−1^ are ascribed to absorbed water. After L-lysine was grafted onto GO, the CH/CH_2_ stretching vibration of the obtained Lys-GO material can be seen at about 2923 cm^−1^. More significantly, a new peak at 1574 cm^−1^ indicated N–H (amide-II bands). The peaks at 1208, 1574 and 836 cm^−1^ are attributed to characteristic amide bonds [16,23]. They are the result of the amidation reaction between the –COOH groups of GO and the amine groups of L-lysine. These results indicate that L-lysine has been successfully anchored to the GO sheets.

**Figure 2:**
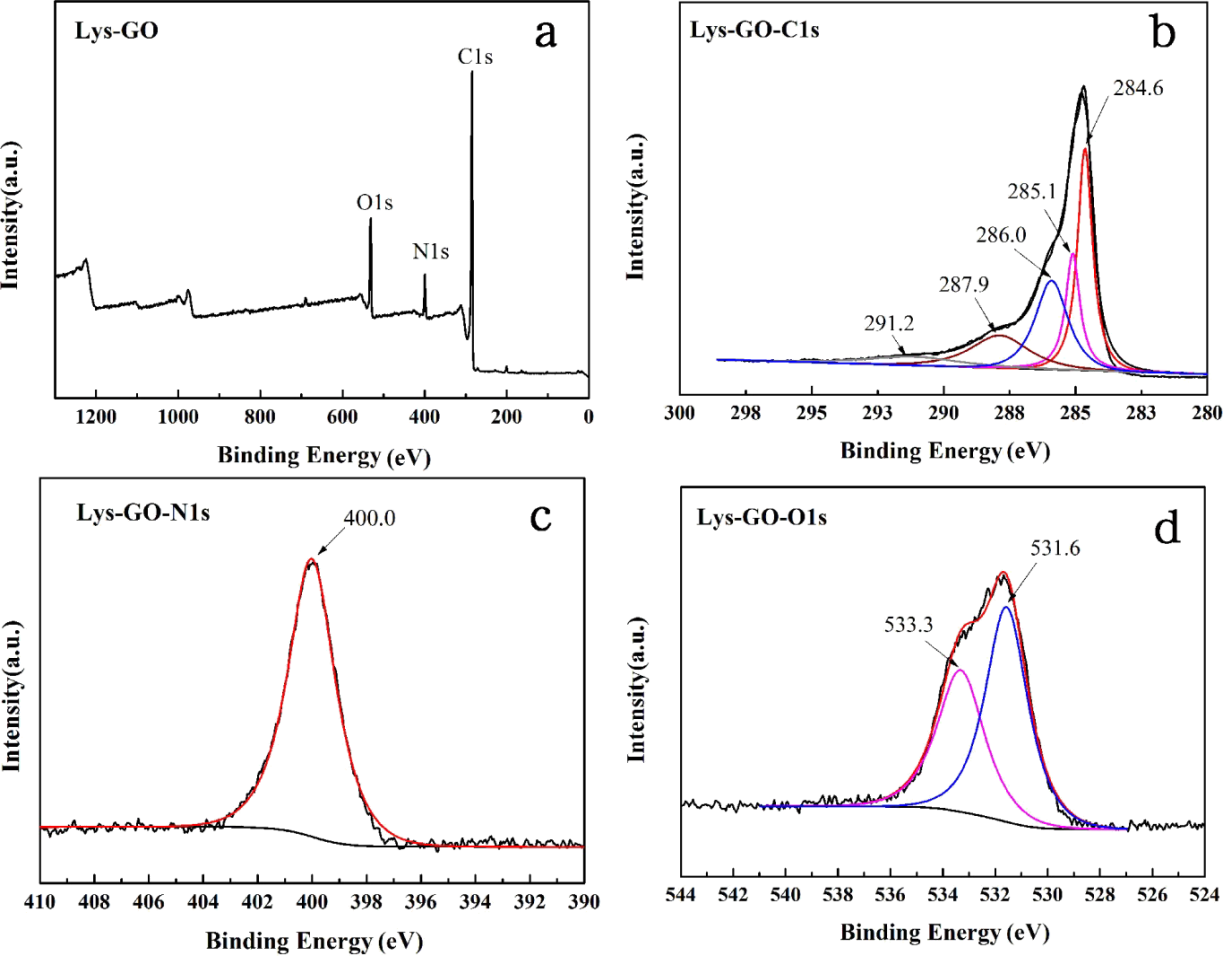
(a) XPS survey spectrum of Lys-GO, (b) C 1s XPS spectrum of Lys-GO, (c) N 1s XPS spectrum of Lys-GO and (d) O 1s spectrum of Lys-GO.

Next, the chemical states and components of the Lys-GO material were measured by XPS analysis (Figure 2). The full XPS spectrums in the binding energy range of 0–1300 eV of Lys-GO are shown in Figure 2a, which could identify the surface element composition clearly. Only carbon (C 1s), oxygen (O 1s) and nitrogen (N 1s) peaks were recorded in the XPS survey spectrum of Lys-GO. This result illustrated that L-lysine was grafted onto the GO surface successfully. In addition, C 1s, N 1s and O 1s peaks were also analyzed to explain the chemical composition of Lys-GO. The C 1s spectrum of Lys-GO was deconvoluted into five main peaks at 284.6, 285.3, 286.0, 287.9, and 291.2 eV in Figure 2b, which were attributed to C–C/C=C, C–N, C–O, C=O, and O–C=O, respectively [24]. The N 1s spectrum of Lys-GO showed only one peak (Figure 2c), which corresponds to the nitrogen atoms of the amide group (N–C=O) at 400.0 eV. The O 1s spectrum was divided into two peaks at 531.6 eV and 533.3 eV (Figure 2d), which indicated different oxygen functional groups in the Lys-GO sample, and represented the O 1s in amide functional group (N–C=O) and the hydroxyl group (C–OH), respectively. These results showed that L-lysine can be easily grafted on the surface of graphene oxide by a simple chemical method. This process is much more economical than other methods, and can be produced on a large scale.

**Figure 3:**
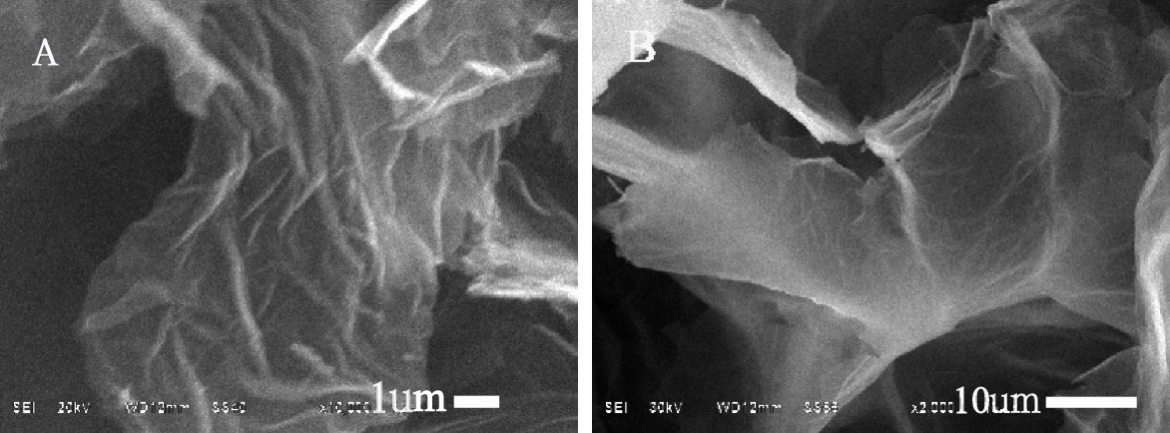
SEM images of Lys-GO (A) and GO (B).

Finally, the morphology of as-prepared graphene materials was characterized by SEM (Figure 3). GO exhibits a structure of the ultra-thin layers with little wrinkles in (Figure 3b). More wrinkled and folded nanosheets of Lys-GO can be clearly seen in Figure 3a. These results showed that the as-prepared Lys-GO may have more vacancy defects and functional groups by the chemical modification, which may lead to an enhanced performance in the adsorption of pollutants.

### Effect of the pH value on the adsorption of MB and Cu^2+^

**Figure 4:**
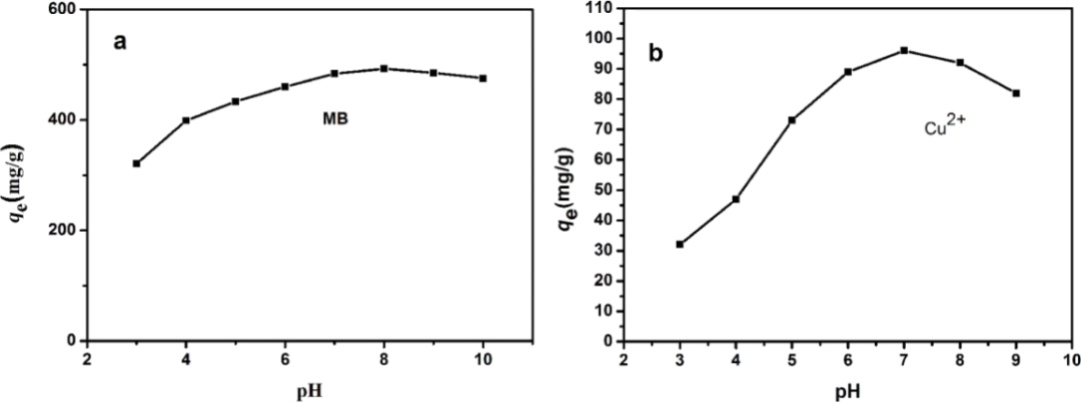
Effect of solution pH value on the adsorption of (a) MB (*C*_0_ = 500 mg/L) and (b) Cu^2+^ (*C*_0_ = 100 mg/L) on Lys-GO.

In order to identify the adsorption capability of Lys-GO for MB and metal ions, a series of batch adsorption experiments were conducted. First, the effect of the pH value on MB and Cu^2+^ adsorption was studied. The initial pH value as an important parameter may control the adsorption process, particularly the adsorption capacity, because the adsorption equilibrium changes with different pH values. Hence, the variation of MB and Cu^2+^ adsorption on Lys-GO under different pH conditions was investigated (Figure 4).

The amount of MB adsorbed on Lys-GO increases with increasing pH values until pH 8 and then decreases again slowly (Figure 4a). For Cu^2+^, adsorption amount increases with increasing pH value from pH 3 to pH 7 (Figure 4b). When the pH value is increased further, the amount of adsorbed Cu^2+^ is reduced again. The optimal pH values for the adsorption of MB and Cu^2+^ on Lys-GO were 8.0 and 7.0, respectively. The effect of the pH value on the adsorption of MB and Cu^2+^ can be attributed to the form of ionic species in the solutions [25–26]. The higher or lower the pH value, the more anions or cations will exist in the solution, which might be bad for the removal of MB and Cu^2+^.

### Adsorption kinetics

**Figure 5:**
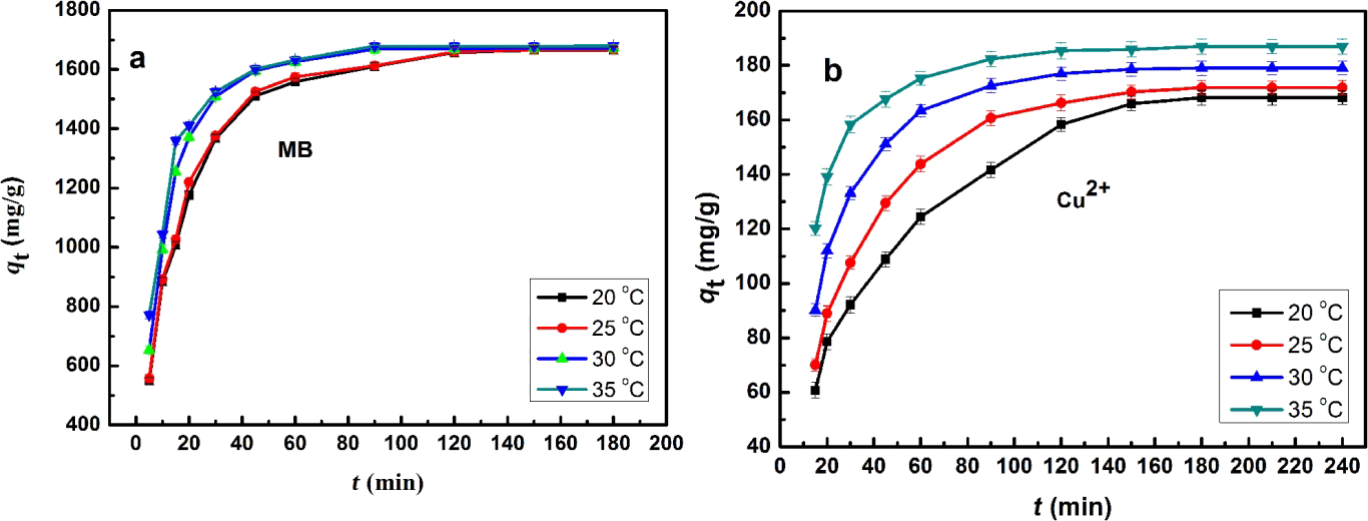
The adsorption of (a) MB (*C*_0_ = 1800 mg/L, pH 8.0) and (b) Cu^2+^ (*C*_0_ = 200 mg/L, pH 7.0) on Lys-GO.

To better understand the processes and mechanisms of adsorption, the adsorption kinetics was investigated. In Figure 5, the adsorption of MB and Cu^2+^ on Lys-GO as a function of the time at different temperatures are shown.

From Figure 5, it can be observed that the kinetic equilibrium for the adsorption of MB on Lys-GO was reached after 120 min, while that of Cu^2+^ is reached after 180 min. Moreover, the equilibrium adsorption capacity of MB and Cu^2+^ on Lys-GO increases slightly with the increase of temperature. A similar character can be seen for all curves. The adsorption process is very fast before 50 min, and then slows down until the equilibrium was reached. The whole at 20 °C can be presented by the formula: *y* = *a*·ln(*x*) + *b* (*R*^2^ = 0.94). To further investigate the adsorption process, the experimental adsorption kinetic data were analyzed using two conventional kinetic models (linear pseudo-first-order and linear pseudo-second-order).

The linear pseudo-first-order kinetic model can be expressed as:


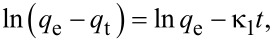


where *q_e_* and *q*_t_ are the adsorbed amounts (mg/g) of MB or Cu^2+^ at equilibrium and at different times *t*, respectively. κ_1_ (min^−1^) is the rate constant of a pseudo-first-order model of adsorption. The values of *q*_e_ and κ_1_ can be calculated from the intercept and slope of the linear plot of ln(*q*_e_ − *q*_t_) as a function of *t*.

The pseudo-second-order model includes all the steps of adsorption including external film diffusion, adsorption, and internal particle diffusion, and can be expressed as:


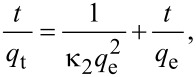


where *q*_e_ and *q*_t_ are defined as above and κ_2_ is the rate constant of the pseudo-second-order model of adsorption (g/mg/min). The slope and intercept of the linear plot of *t*/*q*_t_ against *t* yield the values of *q*_e_ and κ_2_.

**Figure 6:**
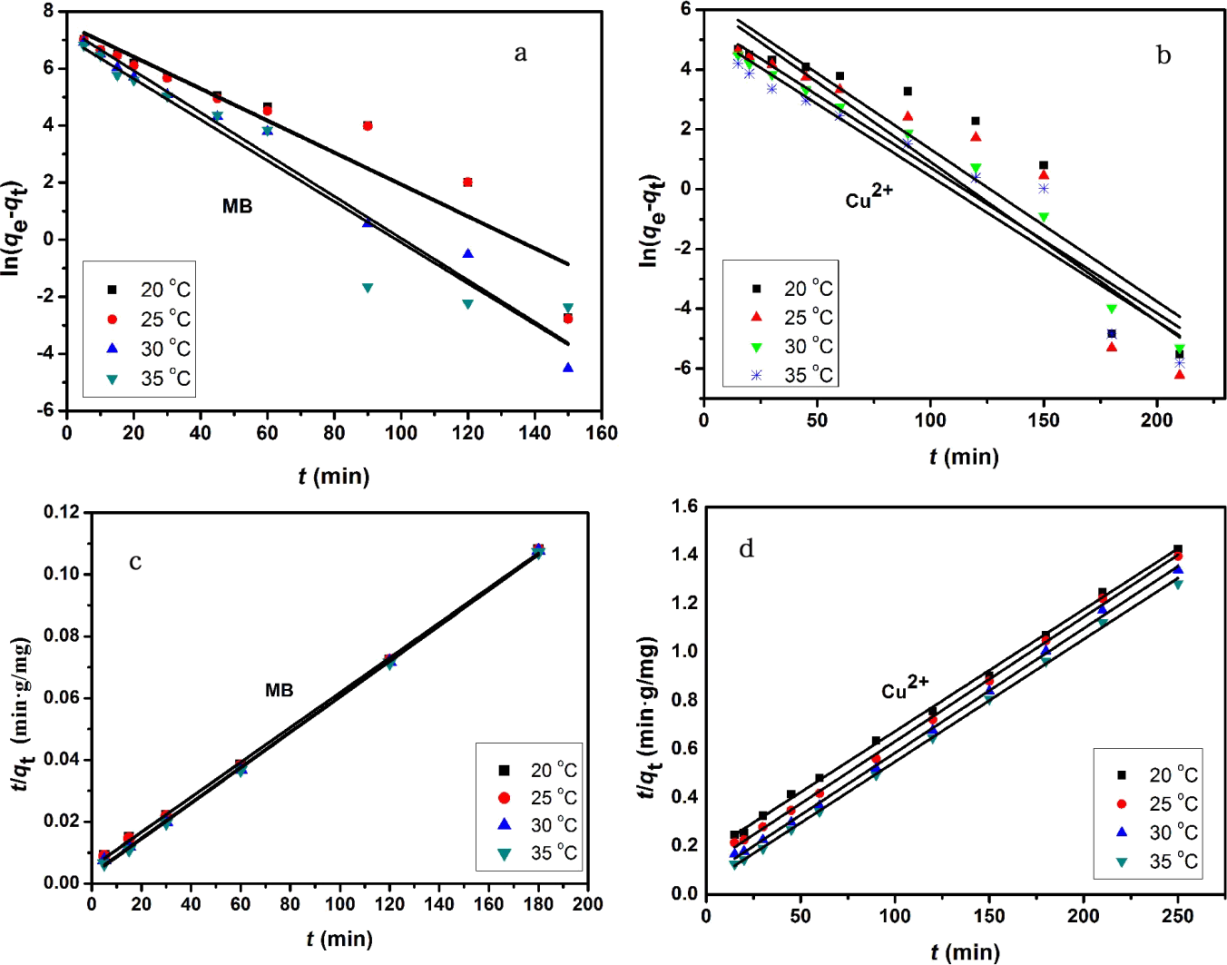
(a,b) Pseudo-first-order and (c,d) pseudo-second-order kinetics models for MB and Cu^2+^ adsorption on Lys-GO (initial conditions: MB 1800 mg/L, solution pH 8.0; Cu^2+^ 200 mg/L, pH 7.0).

The calculated values of *q*_e,cal_, κ_1_, κ_2_, and *R*^2^ from Figure 6 are summarized in Table 1. For the adsorption MB on Lys-GO, the correlation coefficient *R*^2^ for the linear pseudo-second-order model reached up to 0.999, which is much better than that of the pseudo-first-order model. Addtionally, The values of *q*_e,cal_ were also in accordance with the experimental adsorption capacity (*q*_e,exp_) obtained from the pseudo-second-order model. These results indicated that the pseudo-second-order kinetic model may describe the adsorption of MB on graphene. The adsorption kinetic model of Cu^2+^ was very similar to that of MB on Lys-GO, and the linear pseudo-second-order kinetic model fitted the adsorption of Cu^2+^ on Lys-GO.

**Table 1:** The kinetic parameters for the adsorption of MB and Cu^2+^ on Lys-GO^a^.

	MB				Cu^2+^			
temperature	20 °C	25 °C	30 °C	35 °C	20 °C	25 °C	30 °C	35 °C

*q*_e,exp_ (mg/g)	1664.882	1666.358	1670.513	1679.104	168.166	171.832	179.011	186.918

linear pseudo-first-order								
κ_1_ (min^−1^)	5.60·10^−2^	5.59·10^−2^	7.34·10^−2^	7.17·10^−2^	5.08·10^−2^	5.33·10^−2^	4.86·10^−2^	4.84·10^−2^
*q*_e,cal_ (mg/g)	1907.789	1817.014	1620.225	1177.619	611.106	511.563	263.757	193.234
SD	5.195	4.091	2.364	7.465	7.015	6.144	3.069	0.838
*R*^2^	0.8895	0.8882	0.9771	0.9198	0.8445	0.8701	0.9627	0.9134
Linear pseudo-second-order								
κ_2_ (g/mg/min)	5.82·10^−5^	6.18·10^−5^	1.01·10^−4^	1.20·10^−4^	1.48·10^−4^	2.23·10^−4^	3.67·10^−4^	5.91·10^−4^
q_e,cal_ (mg/g)	1772.801	1768.703	1740.508	1738.680	198.807	194.553	194.932	198.020
SD	3.463	3.372	2.789	2.572	1.845	1.589	1.330	1.111
*R*^2^	0.9995	0.9994	0.9993	0.9996	0.9987	0.9988	0.9990	0.9993

^a^SD: standard deviation = [(*q*_e,cal_ − *q*_e,exp_)/(*n* − 2)]^1/2^; *n*: number of data points in the set; κ_1_: rate constant for a pseudo-first-order reaction (min^−1^); κ_2_: rate constant for a pseudo-second-order reaction (g/mg/min); *q*_e_: maximum capacity of adsorption (mg/g).

### MB adsorption isotherms

The adsorption equilibrium isotherm is a key for describing the distribution of the adsorbate molecules between the liquid and the solid phase in equilibrium. Several mathematical models have been widely used to describe equilibrium states of the adsorption of molecules on surfaces. To the best of our knowledge, most of the adsorption systems were generally analyzed by the models of Langmuir or Freundlich. Therefore, the experimental data were also fitted by Langmuir and Freundlich isotherms in this work.

The Langmuir isothermal linear equation is described as


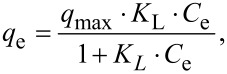


and the Freundlich isothermal linear expression is represented by


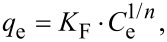


where *C*_e_ (mg/L) is the equilibrium concentration of MB or Cu^2+^ in solution, *q*_e_ (mg/g) is the amount adsorbed on Lys-GO, *q*_max_ (mg/g) is the maximum monolayer adsorption capacity on Lys-GO, *K*_L_ (L/mol) is a coefficient related to the energy of adsorption and is expected to vary with temperature; *K*_F_ (mol^(1 − ^*^n^*^)^·L*^n^*/g) and *n* are constants of the Freundlich isotherm related to the adsorption capacity and adsorption tendency, respectively.

**Figure 7:**
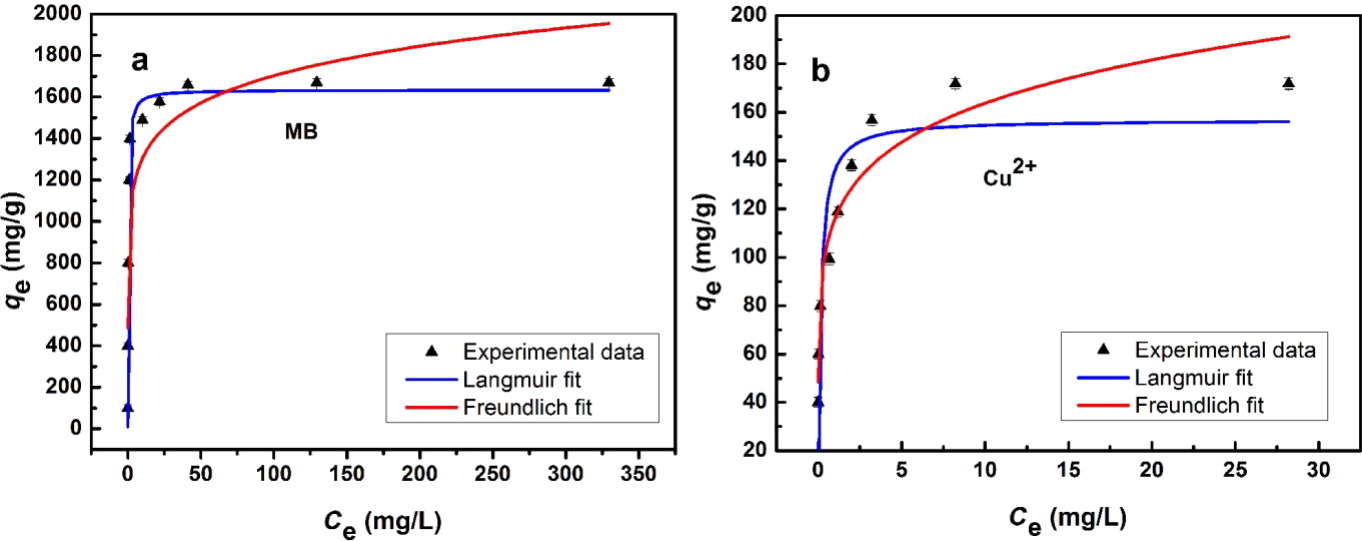
Adsorption isotherms of MB (a) and Cu2+ (b) on Lys-GO.

The adsorption isotherms of MB or Cu^2+^ on Lys-GO are given in Figure 7. The values of calculated Langmuir constants (*K*_L_ and *q*_max_) and Freundlich isotherm constants (*n* and *K*_F_) are listed in Table 2. As seen from Table 2, when the Langmuir model was used to fit the experimental data in Figure 7a, the calculated value the maximum MB adsorption capacity was approximately 1634.36 mg/g, and the determination coefficient *R*^2^ = 0.9879. The value of *R*^2^ (0.7669) obtained from the Freundlich isotherm was much lower than that of the Langmuir isotherm. For the adsorption isotherms of Cu^2+^ on Lys-GO in Figure 7b, the calculated value of the maximum Cu^2+^ adsorption capacity was approximately 156.91 mg/g, and *R*^2^ was equal to 0.6722 using the Langmuir model. As compared with the Langmuir model, the Freundlich model was more suitable for describing the adsorption equilibrium of Cu^2+^ on Lys-GO. Similar result of Cu^2+^ adsorption was reported in [22,24].

**Table 2:** Langmuir and Freundlich isotherm model constants and correlation coefficients for the adsorption of MB and Cu^2+^ on Lys-GO.

adsorbate	Langmuir			Freundlich		
	*q*_max_ (mg/g)	*K*_L_ (L/mol)	*R*^2^	*n*	*K*_F_ (mol^(1−^*^n^*^)^·L^n^/g)	*R*^2^

MB	1634.362	3.23	0.9879	8.64	999.02	0.7669
Cu^2+^	156.908	6.49	0.6722	6.67	115.90	0.9281

The removal of MB and Cu^2+^ with the help of other materials has been extensively studied. The adsorption capacities of other adsorbents were compared in Table 3. Obviously, the adsorption performance of Lys-GO for MB and Cu^2+^ are much better than those of the other previously reported materials. The excellent adsorption performance of Lys-GO might be attributed to two factors: 1) Grafting L-lysine on the GO can improve the affinity for MB and Cu ions because more functional groups are available. 2) Strong π–π interactions and vacancy defects are good for enhancing the adsorption capacity of graphene materials. These results indicated that Lys-GO material has great potential application in treating wastewater.

**Table 3:** Comparison of MB and Cu^2+^ adsorption capacities on various materials.

adsorbate	type of adsorbents	*q*_max_ (mg/g)	reference

MB	Mt-SB12	254	[27]
	agar/graphene oxide (AGO)	578	[28]
	graphene oxide/calcium alginate (GO/CA)	181.81	[29]
	GO/MgO NCs	833	[30]
	magnetic graphene sponge (Fe_3_O_4_-GS)	526	[31]
	Lys-GO	1679.1	this work
Cu^2+^	Mt-SB12	10.2	[27]
	magnetic cassava residue microspheres (MCRS)	110.5	[32]
	PAN-kapok hollow microtubes	90.1	[33]
	silico-manganese nanohybrid adsorbent (SMNA)	40–88	[34]
	GO1	91.6	[35]
	Lys-GO	186.9	this work

## Conclusion

A novel amino acid–graphene composite material was synthesized using graphene oxide (GO) and L-lysine. The interfacial property of GO can be improved by the chemical modification. The as-prepared Lys-GO material as an adsorbent was systematically evaluated with respect to the removal methylene blue (MB) and Cu^2+^ from simulated wastewater. Compared to GO, the performance and adsorption capacity of the modified Lys-GO material was significantly improved. The maximum adsorption capacity for MB and Cu^2+^ was 1679.1 mg/g and 186.9 mg/g, respectively. The kinetics of adsorption followed well the linear pseudo-second-kinetic model. For the Lys-GO material, the isotherm results showed that MB adsorption fitted the Langmuir isotherm model, while Cu^2+^ fitted the Freundlich model. The adsorption capacity of MB and Cu^2+^ on Lys-GO prepared from GO was much better than that of other materials, such as GO/MgO NCs or GO with different defects. The functionalized graphene material may be a promising candidate for the removal of environmental pollutants.

## Experimental

### Materials and instrumentation

Graphite powder was purchased from Shanghai Huayi Company (Shanghai, China). KMnO_4_, NaNO_3_, H_2_SO_4_ (98%) and HCl (36–38%) were obtained from Sinopharm Chemical Reagent Co. Ltd. (Shanghai China). The deionized (DI) water used throughout all experiments was purified to 18.2 MΩ·cm with the Millipore system.

Lys-GO was characterized by scanning electron microscopy (SEM, AJEOL JSM-6510LV, JAPAN) and X-ray photoelectron spectroscopy (XPS, Kratos XSAM-800, UK). Fourier-transform infrared (FTIR) spectroscopy was performed on a Perkin-Elmer model 2000 FTIR spectrophotometer using the Spectrum v. 2.00 software package. MB solution was analyzed using a UV spectrophotometer (Shimadzu, UV-2550) by monitoring the absorbance changes at the wavelength of maximum absorbance (664 nm). Cu^2+^ concentration was analyzed using atomic absorption spectrometry at 324.8 nm (AAanalyst300, Perkin-Elmer).

### Synthesis of the Lys-GO hybrid

**Scheme 1:**
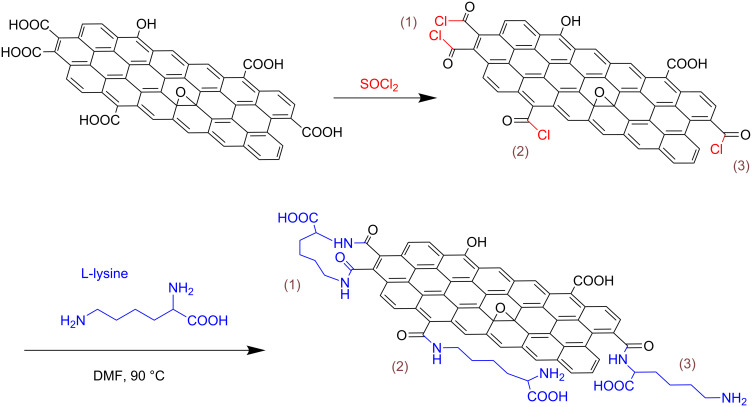
Synthesis of Lys-GO.

Typically, GO (30 mg) was treated with SOCl_2_ (20 mL) in the presence of 0.5 mL of dry *N*,*N*-dimethylformamide (DMF) in a 50 mL round-bottomed flask and heated to 70 °C for 24 h, using an absorption device of neutralization tail gas. After completion of the reaction, the solvent was evaporated at 100 °C and the solid was washed by anhydrous tetrahydrofuran (THF). The obtained product was reacted with L-lysine (2.0 mmol) in anhydrous DMF (20 mL) at 90 °C for 12 h. The L-lysine modified graphene sheets were obtained by filtration and washed by 5 wt % NaHCO_3_, deionized water, and ethanol to remove the unreacted amino acids, respectively. Finally, the sample was dried under vacuum at 50 °C. The whole route of synthesis is shown in Scheme 1.

### Adsorption kinetics

The adsorption as a function of the time was studied to obtain the kinetics information. 10 mg absorbent and 10 mL of MB or Cu^2+^ solution of known concentration were transferred in flask and shaken at four different temperatures (20, 25, 30, and 35 °C). The solutions were collected at different time points (5–250 min), and then centrifuged at 12,000 rpm for 10 min. The liquids were analyzed for MB or Cu^2+^ concentration measurements. The obtained data in batch mode studies were used to calculate for each sample of Cu^2+^ or MB as follows:


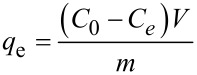


where *q*_e_ is the equilibrium adsorption capacity of Cu^2+^ or MB on Lys-GO (mg/g), *V* is the sample volume (L), *C*_0_ and *C*_e_ are the initial and equilibrium concentration of Cu^2+^ or MB (mg/L) respectively, and *m* is the weight of Lys-GO (g).

### Adsorption isotherms

To quantify the adsorption isotherms, 10 mL of MB or Cu^2+^ solution with different initial concentrations was added to 10 mg Lys-GO, and shaken for 250 min at 303 K. The supernatant was collected by centrifugation. The concentration of remnant MB was measured by UV spectrophotometry, referring to a standard curve. The concentration of remnant Cu^2+^ was analyzed using atomic absorption spectrometry at 324.8 nm.
